# The Role of Muscle microRNAs in Repairing the Neuromuscular Junction

**DOI:** 10.1371/journal.pone.0093140

**Published:** 2014-03-24

**Authors:** Gregorio Valdez, Mary P. Heyer, Guoping Feng, Joshua R. Sanes

**Affiliations:** 1 Department of Molecular and Cellular Biology and Center for Brain Science, Harvard University, Cambridge, Massachusetts, United States of America; 2 Virginia Tech Carilion Research Institute, Virginia Tech, Roanoke, Virginia, United States of America; 3 Department of Neurobiology, Duke University Medical Center, Durham, North Carolina, United States of America; 4 Department of Molecular Therapeutics, The Scripps Research Institute Florida, Jupiter, Florida, United States of America; 5 McGovern Institute for Brain Research, Department of Brain and Cognitive Sciences, Massachusetts Institute of Technology, Cambridge, Massachusetts, United States of America; Georgia Regents University, United States of America

## Abstract

microRNAs have been implicated in mediating key aspects of skeletal muscle development and responses to diseases and injury. Recently, we demonstrated that a synaptically enriched microRNA, miR-206, functions to promote maintenance and repair of the neuromuscular junction (NMJ); in mutant mice lacking miR-206, reinnervation is impaired following nerve injury and loss of NMJs is accelerated in a mouse model of amyotrophic lateral sclerosis (ALS). Here, we asked whether other microRNAs play similar roles. One attractive candidate is miR-133b because it is in the same transcript that encodes miR-206. Like miR-206, miR-133b is concentrated near NMJs and induced after denervation. In miR-133b null mice, however, NMJ development is unaltered, reinnervation proceeds normally following nerve injury, and disease progression is unaffected in the SOD1(G93A) mouse model of ALS. To determine if miR-206 compensates for the loss of miR-133b, we generated mice lacking both microRNAs. The phenotype of these double mutants resembled that of miR-206 single mutants. Finally, we used conditional mutants of Dicer, an enzyme required for the maturation of most microRNAs, to generate mice in which microRNAs were depleted from skeletal muscle fibers postnatally, thus circumventing a requirement for microRNAs in embryonic muscle development. Reinnervation of muscle fibers following injury was impaired in these mice, but the defect was similar in magnitude to that observed in miR-206 mutants. Together, these results suggest that miR-206 is the major microRNA that regulates repair of the NMJ following nerve injury.

## Introduction

Aging and motor neuron diseases such as amyotrophic lateral sclerosis (ALS) significantly affect the structure and molecular composition of the neuromuscular junction [1]–[11]. In these conditions, the postsynaptic apparatus fragments and axons withdraw, leaving muscle fibers denervated. The inability of aged and ALS-affected motor axons to quickly reinnervate muscle fibers following bouts of degeneration [12], [13] contributes to the atrophy of muscle fibers and death of motor neurons. Thus, identification of factors that promote regeneration of NMJs could aid in the development of approaches for maintaining motor function in old age or disease. Recent evidence that exercise and a calorically restricted diet can reduce and even reverse age-associated structural changes at the NMJ [6] encourages a search for such mechanisms.

microRNAs (miRNAs) have been implicated in modulating stress responses in numerous cell types, including neurons and muscle cells [14], [15]. miRNAs are ∼22 nucleotide small RNAs that bind to complementary regions on numerous mRNAs, thereby promoting their degradation and inhibiting translation [16], [17]. A single miRNA therefore has the ability to modulate and coordinate multiple signaling cascades [18]. Skeletal muscles express a rich assortment of miRNAs, including the highly conserved miR-206/miR-1 and miR-133a/b families of miRNAs [19], [20]. Interestingly, miR-206 and miR-133b are present in a single transcript called 7H4 that is concentrated in synapse-rich regions of muscles fibers beneath the postsynaptic membrane at the NMJ [21]. To date, functional analysis of 7H4 has focused on miR-206. Studies *in vitro* and *in vivo* have shown that miR-206 is involved in formation of myofibers, differentiation of satellite cells, and formation of new NMJs following nerve injury [13], [22]–[26]. Levels of miR-206 are upregulated following denervation and in mouse models of muscular dystrophy and ALS; in the absence of miR-206, reinnervation is slowed following nerve injury and disease progression is accelerated in SOD1(G93A) mice [13], [27]. These results suggest that muscles upregulate miR-206 in a futile attempt to promote reinnervation and resist disease progression [13].

Our aim in this study was to determine whether miR-206 is unique in its ability to promote reinnervation or whether other miRNAs such as miR-133b play similar or even more powerful roles. miR-133b was an ideal first candidate due to its location in the 7H4 transcript. We first confirmed that miR-133b, like miR-206, is synaptically enriched and highly upregulated in muscle following motor nerve injury. We then proceeded in three steps: first analyzing mice lacking miR-133b, then mice lacking both miR-133b and miR-206, and finally mice lacking most miRNAs in postnatal muscle fibers. Deletion of miR-133b had no significant effect on reinnervation following acute injury or on disease progression in SOD1(G93A) mice, while effects of deleting both miR-133b and miR-206 or most miRNAs resembled those of deleting miR-206 alone. Together, our findings suggest that miR-206 plays a unique role in stress responses at the NMJ.

## Results

### Localization and expression pattern of miR-133b

**Figure 1:**
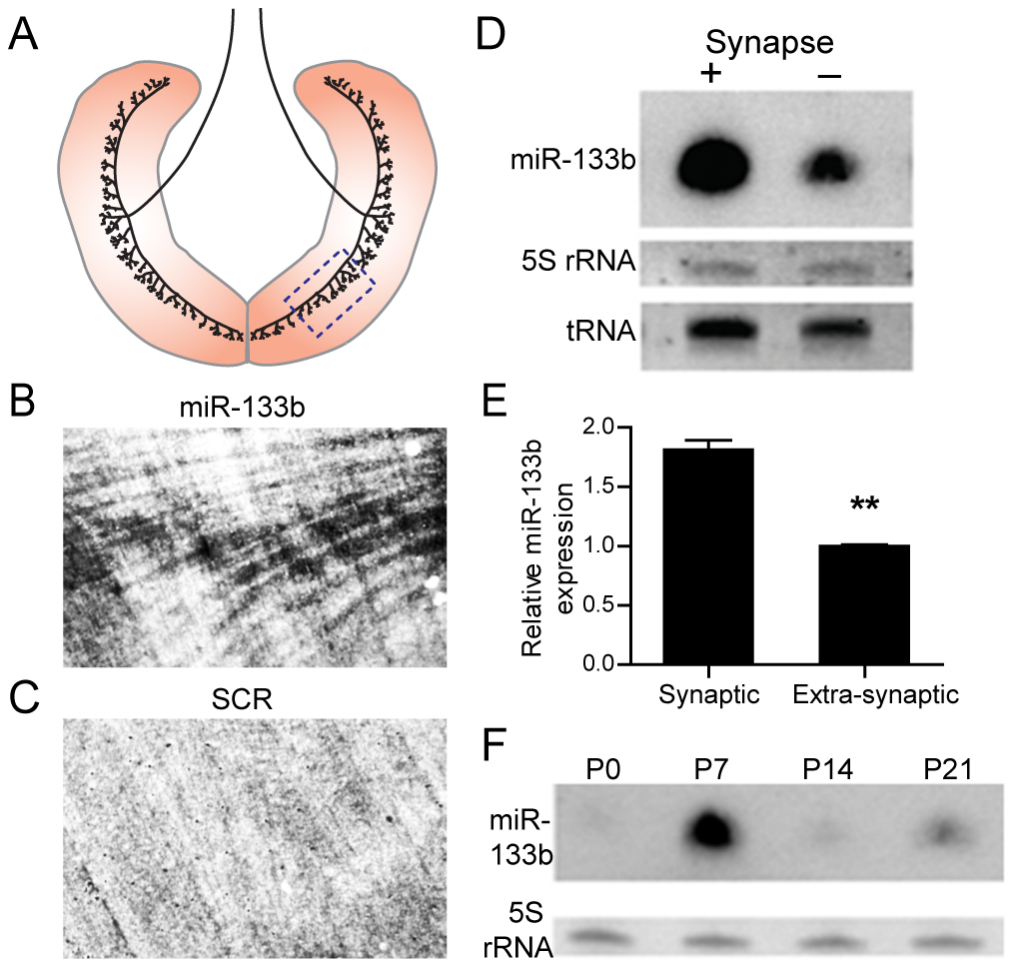
Expression of miR-133b at the neuromuscular junction. (**A**) Schematic of phrenic nerve innervation and location of NMJs in the mouse diaphragm. Dotted box (A) indicates the region examined using *in situ* hybridization (B, C). (**B, C**) Whole-mount miRNA *in situ* hybridization of P0 diaphragm with antisense (B) but not scrambled (C) DIG-labeled LNA probes shows restriction of miR-133b signal to the central synaptic band. (**D**) Northern blot analysis of mature miR-133b expression in synaptic (+) and extrasynaptic (−) regions of P21 mouse diaphragm. (**E**) Intensities of bands in (D), normalized to tRNA, show synaptic enrichment of miR-133b. Error bars indicate SEM. (**F**) Northern blot analysis of mature miR-133b expression during development of postnatal tibialis anterior muscle.

miR-206 levels peak early in postnatal life, increase following denervation, and are higher in synaptic than extrasynaptic regions of mature muscles [13]. miR-133b and miR-206 are present in the same transcript [21], suggesting that they are co-regulated [13], [28]. However, independent control of the expression of these two miRNAs was documented in a recent study [29]. To determine if miR-133b is also synaptically enriched, we analyzed its expression in micro-dissected synaptic and extrasynaptic regions from 21 days old diaphragm muscles. We used animals that express green fluorescence protein (GFP) in motor axons [30] to distinguish synaptic from extrasynaptic regions, and probed northern blots with digoxigenin (DIG)-end-labeled LNA probes that anneal with mature miR-133b [31], [32]. We also used *in situ* hybridization to visualize miR-133b in the end-plate band region of the diaphragm muscle. This synaptic region is easily determined in the relatively thin, flat diaphragm by noting the position of the phrenic nerve that runs centrally in each hemidiaphragm and perpendicularly to the muscle fibers (Fig. 1A) [21], [33]. Both methods demonstrated that miR-133b levels are significantly higher in synaptic regions compared to non-synaptic regions of the same muscle (Fig. 1A–E). We then analyzed muscles from mice of several ages, and found that miR-133b increases during the first postnatal week, then declines in adulthood (Fig. 1F). In all of these respects, the localization and expression of miR-133b resembles that shown previously for 7H4, and miR-206 [13], [21].

### Role of miR-133b at the NMJ

**Figure 2:**
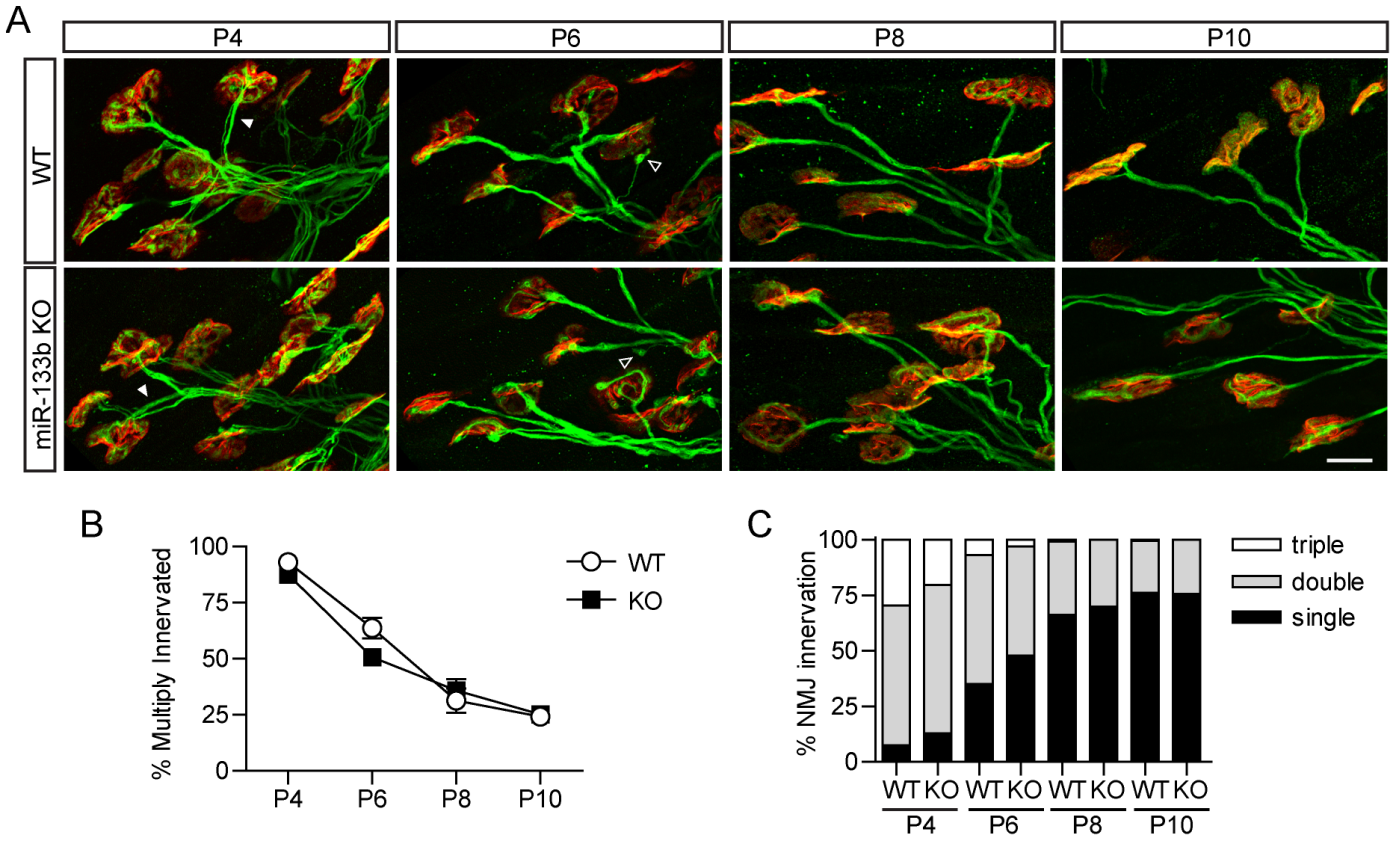
Normal NMJ development in miR-133b knockout mice. (**A**) Immunofluorescence staining of axonal neurofilaments and vesicular synaptophysin (green) and BTX staining of postsynaptic nAChRs (red) to visualize axons innervating synaptic sites. Filled white arrowheads, NMJs with multiple axon innervation; empty arrowheads, retraction bulbs. Scale bar  = 20 μm. (**B**) The proportion of sternomastoid NMJs with multiple innervation decreases at a similar rate in control and knockout mice. (**C**) Proportion of developing sternomastoid NMJs with single, double or triple innervation is similar in control and knockout mice.

We used miR-133b mutant mice [34] to seek roles of this miRNA in neuromuscular development. Homozygous null mutants are outwardly normal and fertile. Sternomastoid muscles at different stages of development were stained with antibodies against neurofilaments and synaptophysin to visualize the axon and nerve terminal, respectively, and with fluorescently tagged alpha-bungarotoxin (f-BTX) to label acetylcholine receptors (AChRs) in the postsynaptic membrane. High resolution confocal images revealed no apparent difference between mutants and controls in the size or shape of the NMJ (Fig. 2A). We also determined the number of axons present at the NMJ during the first two postnatal weeks; during this period, individual NMJs, which are multiply innervated at birth, lose all inputs save one [1]. Synapse elimination occurred at the same rate in controls and miR-133b mutants (Fig. 2B–C). Hence, miR-133b is dispensable for development of the NMJ.

### Reinnervation and disease progression in miR-133b knockout mice

**Figure 3:**
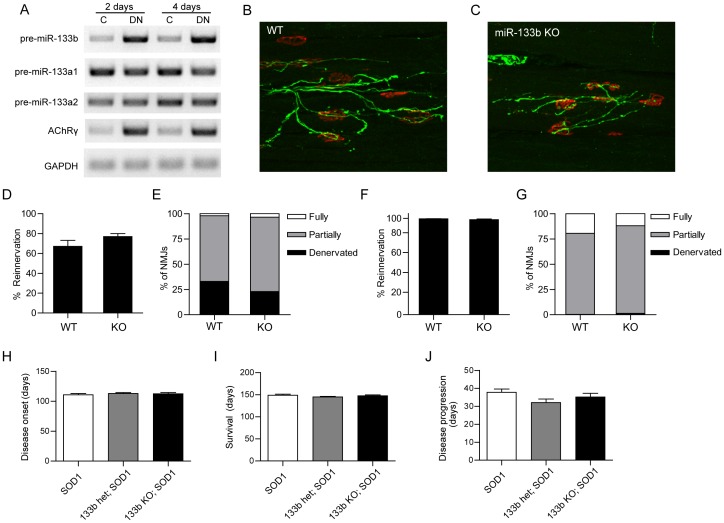
MiR-133b does not regulate muscle reinnervation or ALS disease progression. (**A**) Semi-quantitative RT-PCR of cDNA from control or denervated hindlimb muscle 2 and 4 days after unilateral sciatic nerve cut. Levels of pre-miR-133b and AChRγ increase dramatically in denervated muscle, while levels of pre-miR-133a-1, pre-miR-133a-2 and GAPDH are unchanged, suggesting differential regulation of miR-133a and miR-133b. (**B–E**) Analysis of muscle reinnervation in tibialis anterior muscle from control (B) and miR-133b null mice (C) 3 weeks following nerve cut. (**D**) Percentage of tibialis anterior NMJs that were reinnervated. (**E**) Percentage of NMJs that were denerverated, partially reinnervated, or fully reinnervated. (**F,G**) Analysis of sternomastoid muscle reinnervation 9 days following accessory nerve crush. At least 6 mice were analyzed and 200 NMJs were examined per animal. Error bars indicate SEM. Scale bar  = 20 μm. (**H–J**) In the SOD1-G93A mouse model for ALS, loss of miR-133b does not exacerbate symptoms; disease onset (H), survival rate (I), and disease progression (J) are unchanged in the absence of miR-133b. Data were obtained from: 8 female, 8 male SOD1G93A; 10 female, 8 male miR-133b+/−;SOD1G93A; and 6 female, 9 male R-133b−/−;SOD1G93A mice. Error bars indicate SEM.

To assess the role of miR-133b in reinnervation, we first mapped its expression following transection of the sciatic nerve. Control and denervated hindlimb muscles were harvested 2 and 4 days after denervation, and miR-133b precursor levels were assayed by semiquantitative RT-PCR. We used the AChRγ subunit, which is known to be induced following denervation [35], as a positive control and the metabolic enzyme GAPDH as a negative control. Levels of the miR-133b stemloop and AChRγ subunit mRNA increased within 2 days of denervation. In contrast, levels of pre-miR-133a-1/2 and GAPDH RNAs were not detectably changed, demonstrating differential regulation of miR-133a and miR-133b (Fig. 3A). Thus, we also find that miR-133b expression is increased in denervated skeletal muscles as previously demonstrated[13], .

The induction of miR-133b after denervation is consistent with the idea that it, like miR-206, may be involved in promoting muscle reinnervation. To test this possibility, we injured motor axons by cutting the sciatic nerve and examined reinnervation of muscles in mice lacking miR-133b and in littermate controls. After 3 weeks, the extent of reinnervation in the tibialis anterior muscle did not vary detectably between controls and mutants (Fig. 3B–E). We also assayed reinnervation in the sternomastoid muscle after crushing the accessory nerve, a manipulation that leads to prompt reinnervation. We found no difference in the rate of muscle reinnervation 9 days post-injury (Fig. 3F–G). These results indicate that, unlike miR-206, miR-133b does not promote regeneration of NMJs.

In the SOD1(G93A) mouse model of amyotrophic lateral sclerosis (ALS), miR-133 as well as miR-206 are upregulated at symptomatic stages [13]. Our previous analysis showed that deletion of miR-206 accelerates disease progression in this model; we suggested that upregulation of miR-206 is part of a futile attempt by muscle to maintain innervation or enhance reinnervation [13]. To ask whether miR-133b plays a similar role, we crossed miR-133b mutants to SOD1(G93A) mice and monitored disease onset, survival, and disease progression. We observed no significant difference between SOD1(G93A) mice and SOD1(G93A); miR-133b^−/−^ mice in any of these parameters (Fig. 3H–J). Thus, miR-133b is not required to maintain or restore NMJs following acute nerve injury or in a motor neuron disease.

### Generation of miR-206 and miR-133b double knockout mice

**Figure 4:**
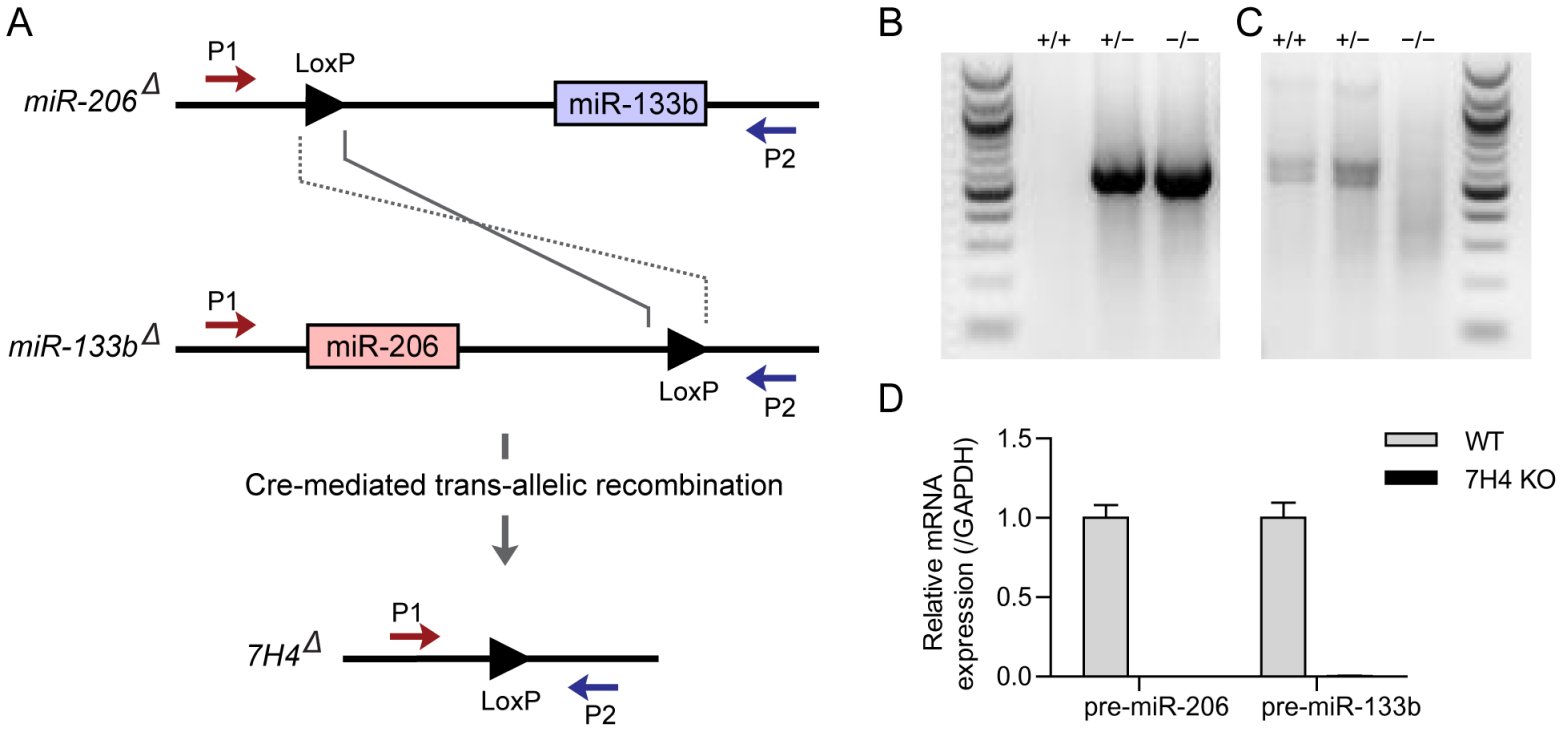
Generation and analysis of miR-206 and miR-133b double knockout mice. (**A**) *Trans*-allelic targeted meiotic recombination was used to generate mice lacking both miR-206 and miR-133b. MiR-206 and miR-133b heterozygous mice each containing a loxP site in place of the miRNA stemloop were bred together and with mice expressing Cre recombinase in the germline. Zygotes produced from sperm that underwent *trans*-allelic recombination contained one chromosome lacking miR-206 and miR-133b and one chromosome with a miR-206 and miR-133b duplication. These animals were then bred to obtain miR-206 and miR-133b double knockout mice, i.e. 7H4 knockout mice. P1, forward primer upstream of miR-206. P2, reverse primer downstream of miR-133b. (**B**) PCR using P1 and P2 primers (in A) gives a detectable product (550 bp) only in 7H4 heterozygous and knockout mice, demonstrating that the 7H4 genomic region containing the miR-206 and miR-133b stem loops is completely missing from the 7H4 null allele. (**C**) PCR using primers specific for the miR-133b allele yields a 600 bp band only when the WT allele is present, but no band for the 7H4 null allele. (**D**) Quantitative RT-PCR for the stemloop regions of miR-206 and miR-133b. As expected, miR-206 and miR-133b are absent in 7H4 knockout mice.

miR-206 and miR-133b are encoded at the 5′ and 3′ end, respectively, of a transcript called 7H4 [21], [36], explaining their similar expression pattern. Although their seed sequences are distinct, several transcripts are predicted to be targeted by both miR-206 and miR-133b (Table S1). To examine the possibility of functional redundancy between miR-206 and miR-133b, we generated mice lacking both miRNAs. miR-206 and miR-133b are separated by only 3.7 kb, making it highly unlikely to generate a double mutant by ordinary meiotic recombination. In contrast, *trans*-allelic cre-mediated meiotic recombination between loxP sites is capable of generating interchromosomal deletions and duplications at a single locus during pairing of homologous chromosomes at the first meiotic division [37], [38]. We therefore used this method (Fig. 4A). miR-206 and miR-133b heterozygous mice, each containing a loxP site in place of the miRNA stem loop, were bred together and the trans-heterozygotes were then bred with mice expressing Cre recombinase in the germline [13], [34], [39]. We used PCR to identify mice that underwent trans-allelic recombination and therefore contained a chromosome lacking both miR-133b and miR-206 (Fig. 4B–D). We called mice lacking both microRNAs 7H4 mutants.

**Figure 5:**
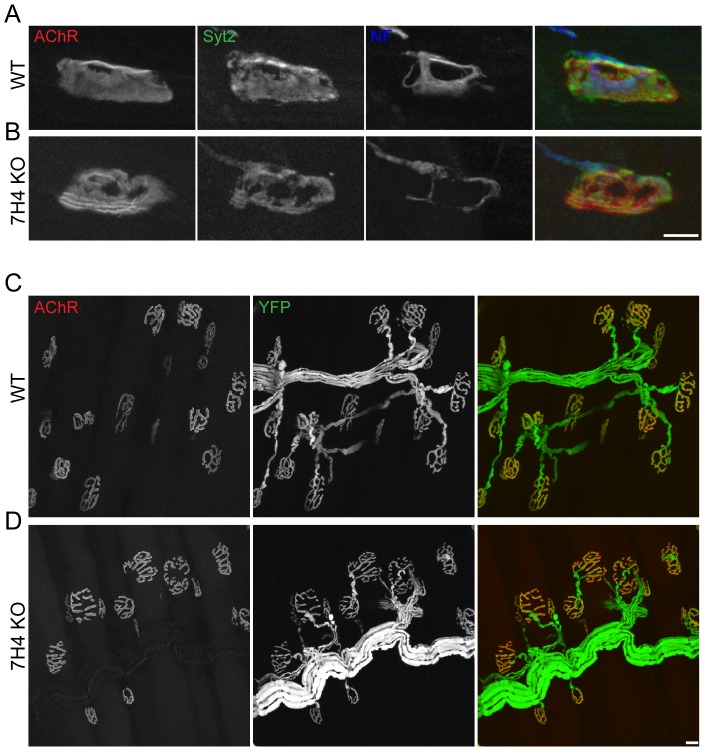
Development of NMJs in 7H4 mice. (**A–D**) Both miR-206 and miR-133b are dispensable for development of the NMJ. There is no obvious difference in the transformation of the postsynapse (stained using f-BTX, red) from a small plaque into a large pretzel between 7H4 knockout (B and D) and control mice of the same age (A and C). The formation of the presynaptic apparatus is also indistinguishable between 7H4 knockout mice and control mice of the same age, visualized using antibodies against synaptotagmin-2, green, and neurofilament, blue, in young animals (A and B) and YFP expressed in motor axons (C and D). Scale bar  = 10 μm for P9 and 20 μm for adult NMJs.

**Figure 6:**
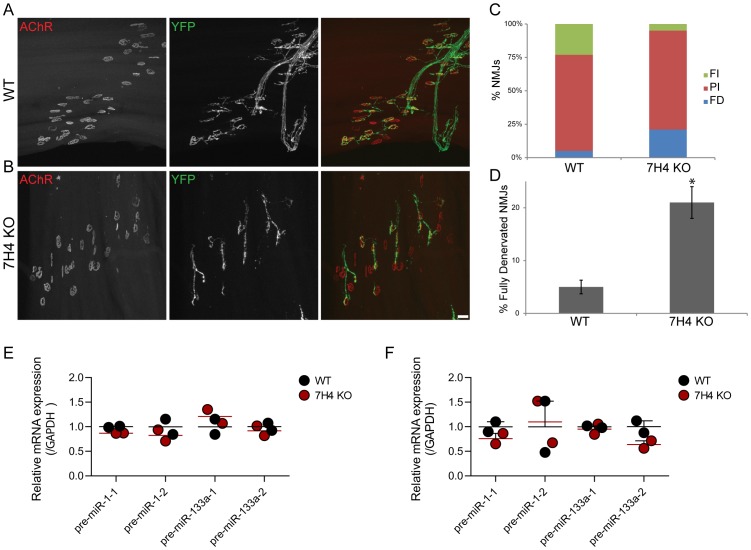
Lack of both miR-206 and miR-133b delays NMJ regeneration. (**A–D**) To determine whether both miRNAs, miR-206 and miR-133b (7H4), act in concert to affect muscle reinnervation, the peroneal nerve was crushed in control (A) and 7H4 knockout mice (B) and reinnervation of the extensor digitorium longus was examined 9 days post injury. In 7H4 muscles, the incidence of partially and completely denervated NMJs is higher than that in muscles from control animals (C, D). At least 6 mice were examined per genotype and 50 NMJs per mouse visualized. FI, fully innervated; PI, partially innervated; FD, fully denervated NMJs. Error bar  =  SEM. P-value (*) <0.02. Scale bar  = 50 μm. (**E, F**) Quantitative mRNA expression of pre-miR-1-1, pre-miR-1-2, pre-miR-133a-1, and pre-miR-133a-2 in EDL (E) and soleus (F) muscle of adult WT (black circles represent individual values and black line the mean) and 7H4 knockout (red circles represent individual values and red line the mean) mice. Gene expression is normalized to Gapdh and results are scaled to the average value of the WT samples.

7H4 mutant mice were viable, fertile and showed no obvious external defects. Their NMJs appeared indistinguishable from those in wild type animals by fluorescence microscopy (Fig. 5A–D). Reinnervation of muscle following nerve crush was delayed in 7H4 mutants as compared to that of control littermates (Fig.6A–D). This result provides further support to the idea that miR-133b is not required for the timely regeneration of NMJs. However, we cannot exclude the possibility that miR-133b acts together with miR-206 to affect reinnervation of muscles. To better assess the role of miR-133b in 7H4 mutants, reinnervation rates would need to be compared between 7H4 and miR-206 mutant mice using the same injury paradigm.

Two loci related to 7H4, which encodes miR-206 and miR-133b, are present in the mouse genome; one encodes miR-1-1 and miR-133a-2 and the other encodes miR-1-2 and miR-133a-1 [20]. We considered the possibility that these genes were upregulated in 7H4 mutants, compensating for loss of miR-133b and miR-206. However, miRNA levels of these two transcripts were not significantly affected by loss of miR-133b and miR-206 in the EDL and the soleus muscle (Fig. 6E–F).

### Deletion of Dicer from postnatal skeletal muscles

**Figure 7:**
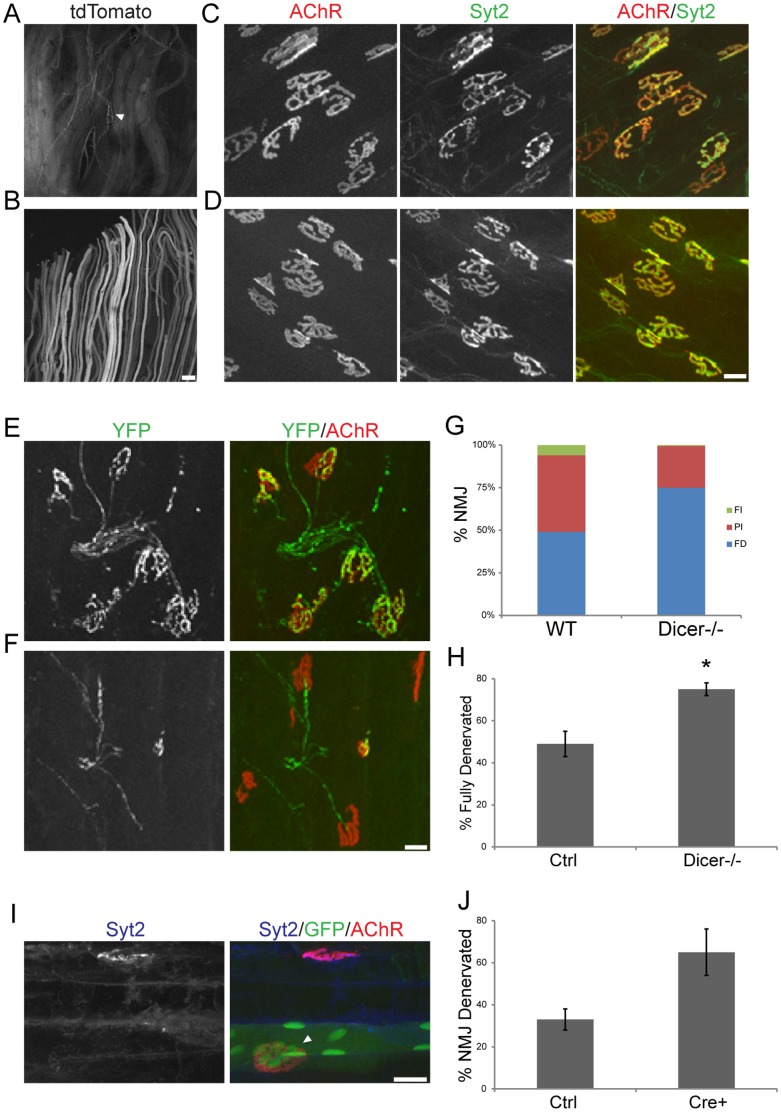
Mature miRNAs are required to promote muscle reinnervation. A tdTomato reporter mouse line was crossed with a mouse expressing Cre recombinase from the Parvalbumin locus. Expression of tdTomato in the EDL, a muscle primarily composed of fast-type muscle fibers, (**B**) and the soleus, a muscle composed mostly of slow-type muscle fibers (**A**). tdTomato is also found in axons of proprioceptive sensory neurons (A, arrow), which express Parvalbumin. Scale bar  = 240 μm. (**D**) NMJs in 1 month-old PV-Cre;Dicer mice are similar to those in control animals (**C**). (**F–H**) In PV-Cre-Dicer mice, muscle reinnervation is delayed compared to controls (**E, G–H**) 7 days post nerve crush. At least 4 mice were examined per genotype and 50 NMJs per mouse visualized. Error bars represent SEM. P-value (*) <0.001. Scale bar  = 20 μm. (**I–J**) Similarly, deletion of Dicer in mature muscle fibers via electroporation of a plasmid expressing Cre recombinase tagged with GFP also slowed down NMJ regeneration. Error bars represent SEM. Scale bar  = 20 μm.

To explore the possibility that other miRNAs are involved in repairing damaged NMJs, we used a conditional allele of Dicer, an enzyme required for generating most miRNAs. Deletion of Dicer from embryonic muscle leads to severe defects in myogenesis and perinatal lethality [40], [41]. We therefore used two methods to delete Dicer postnatally. First, we used a Parvalbumin-Cre (PV-Cre) line [42] that we showed previously acts in postnatal fast (Type IIB) skeletal muscle fibers [43], consistent with the selective expression of this calcium-binding protein in Type IIB muscle fibers [44]. We confirmed that PV-Cre leads to broad activation of a reporter gene in the predominantly fast extensor digitorum muscle but not in the predominantly slow (Type I) soleus muscle (Fig. 7A,B). PV-Cre;Dicer mutants appeared normal at birth, but grew more slowly than littermates and exhibited motor defects as they matured, presumably reflecting the expression of parvalbumin in several neuronal types including proprioceptive sensory neurons, cerebellar Purkinje cells and cortical interneurons [45], [46]. NMJs were not markedly affected in the EDL (Fig. 7C–D), but reinnervation was significantly delayed following denervation in 40 days old mice (Fig. 7E–H). Unfortunately, the small size and other defects precluded direct comparison of these mutants with 7H4 mutants. Thus, it is possible that the delay in reinnervation is more severe in the absence of most miRNAs than in miR-206 and 7H4 mutants.

As a second approach, we deleted Dicer from a few muscle fibers in adult Dicer floxed/floxed animals via electroporation of Cre recombinase tagged with GFP (GFP-Cre). In this experiment, the tibialis anterior muscle was electroporated with GFP-Cre and the peroneal nerve crushed. Nine days after denervation, most GFP-negative (and thus miRNA-positive) muscle fibers were reinnervated. In contrast, most nearby GFP-Cre positive (and thus miRNA-negative) muscle fibers remained denervated (Fig. 7I–J).

## Discussion

The transcriptional profile of skeletal muscle fibers is highly dependent on their state of innervation. Among the best studied are the subunits of the AChR, which are upregulated by denervation, leading to a phenomenon originally called “supersensitivity” [47]. It is generally presumed that increased density of AChRs in the muscle membrane facilitates formation of new NMJs by regenerating axons. We showed recently that a miRNA, miR-206, also promotes reinnervation [13]. Like AChR mRNAs, miR-206 is concentrated near synaptic sites in normal adult muscle and upregulated following denervation. Moreover, reinnervation is delayed in mice lacking miR-206. miR-206 differs from AChRs, however, in that it does not act cell-autonomously, but rather appears to control synthesis and/or release of factors that act on regenerating axons [13]. In this paper, we explored the possibility that other miRNAs act along with miR-206 to maintain and repair the NMJ.

### miR-133b is not required for muscle and NMJ development and repair

miR-133b, like miR-206, concentrates at the NMJ, peaks early in postnatal development, and is induced in unhealthy muscles. However, miR-133b is not required for the development and repair of NMJs. During development, the absence of miR-133b does not alter the maturation of the pre- and post-synaptic apparatus, including the selective aggregation of presynaptic markers at the nerve terminal and the elaboration of the postsynaptic site into a pretzel-like structure. Moreover, lack of miR-133b does not alter the progression of synapse elimination. miR-133b is also dispensable for the maintenance and repair of NMJs. In mice lacking miR-133b, reinnervation of muscle fibers proceeds at the same rate as in control animals. Similarly, loss of this miRNA does not accelerate disease progression in a mouse model of ALS [48].

We next explored the possibility that miR-206 compensates for loss of miR-133b. If this were the case, mice lacking both miRNAs would be expected to exhibit a more severe phenotype than that found in mice only missing miR-206. To test this idea, we generated double mutants lacking both miRNAs as well as the short intervening sequence that separates them. Although NMJs formed and developed normally, reinnervation of denervated muscles was delayed in the absence of both miR-133b and miR-206 (7H4). These findings parallel previous findings in miR-206 single mutants [13]. However, we were unable to determine whether deletion of both miRNAs further delays reinnervation compared to mice lacking only miR-206 because different injury paradigms were used to assess reinnervation in the two mutant strains.

### Roles of synaptic and non-synaptic miRNAs in reinnervation

In contrast to miR-206 and 7H4 knockout mice, early deletion of Dicer prevents muscle development, leading to perinatal death in mice [39],[40]. These results have led to the identification of a myriad of miRNAs involved in the development and response of skeletal muscles to injury and diseases. Potential candidates for mediating such responses include the miR-1 and miR-133a families of miRNAs which are highly similar to miR-206 and miR-133b, respectively. Both sets of miRNAs have been shown to play key roles in myogenesis [13], [18], [19], [21]–[25]. To ask whether these or other miRNAs also contribute to promotion of reinnervation, we depleted a broad repertoire of miRNAs by deletion of Dicer postnatally, thereby avoiding defects in myogenesis and early lethality. We used two methods to delete Dicer postnatally – a Cre line that is expressed in fast-twitch skeletal muscles beginning a few days after birth [42] and electroporation of Cre into adult muscle fibers. In both cases, reinnervation was delayed. Unfortunately, technical issues precluded a direct quantitative comparison of the delay in reinnervation among the different mutants. However, it is worth noting that 24% of PV-Cre;Dicer and 16% of 7H4 mutant muscles remained completely denervated compared to those in littermate controls. Thus, we can conclude that skeletal muscles primarily utilize miR-206 alone or together with miR-133b to maintain and repair the NMJ.

## Materials and Methods

### Source of Mice

The Thy1-YFP [30], miR-133b [33], miR-206 [26], SOD1-G93A [47], PV-Cre [41], Actin-Cre [38], ROSA-CAGS-lox-stop-lox-tdTomato [49] and Dicerf^lox/flox^ conditional mice [40] have been previously described. To generate 7H4, miR-206^−/−^ and miR-133b^−/−^ mice containing LoxP sites facing in the forward direction were crossed with Actin-Cre mice. miR-206^+/−^; Actin-Cre and miR-133b^+/−^;Actin-Cre mice were then crossed to obtain mice lacking both miRNAs (7H4). These mice were then genotyped to determine recombination using primers flanking the 5′ region of miR-206 (Forward primer  =  AGTGTCCATTCATCTCCTACAGCCC) and the 3′ region of miR-133b (Reverse primer  =  CTCGACTGCATTTCCATTGTACTG). Primers that amplify miR-133b were also used to distinguish between heterozygotes and homozygous mice (Forward  =  CAAGCTCTGTGAGAGGTTAGTCAGG; Reverse  =  CTCGACTGCATTTCC ATTGTACTG). Additionally, quantitative real time PCR was carried to validate the absence of pre-miR-206 and pre-miR-133b in these mice; see below. All experiments were carried out under NIH guidelines and animal protocols approved by Harvard University Animal Studies Committee, Virginia Tech Institutional Animal Care and Use Committee and Duke University Institutional Animal Care and Use Committee.

### Acute nerve injury

Surgical procedures were performed on anesthetized 45 to 120 days old mice as previously described [26]. Briefly, mice were anesthetized, the left sciatic nerve was cut and the superficial peroneal nerve or right accessory nerves crushed for 15 or 30 seconds with fine forceps. Animals were sacrificed 7–21 days after denervation to analyze muscle reinnervation. For expression analysis, the sciatic nerve was cut and tibialis anterior muscle isolated 2 to 4 days later.

### Electroporation

DNA was dissolved in normal saline (0.9% NaCl) at a concentration of 4 μg/μl. Mice were anesthetized and their tibialis anterior muscles injected transcutaneously with 25 μL of a 4 U/μL bovine hyaluronidase/saline solution (Sigma). Two hours later, the mice were re-anesthetized, the tibialis exposed, injected with 25 μl of DNA (50 μg) and electroporated. Electroporation was performed with a pair of 0.2 mm diameter stainless steel needle electrodes (Genetronics) held 4 mm apart and inserted on either side of the injection site parallel to the muscle fibers. Six 60 V pulses, each 10 ms in duration, were delivered (BTX electroporator). At appropriate intervals thereafter, muscles were dissected, fixed and imaged.

### Histology

Mice were anesthetized with sodium pentobarbital and perfused transcardially with 4% paraformaldehyde in 0.1 M PBS (PBS; pH 7.4). Muscles were then dissected and post-fixed for an additional 30 minutes. To visualize AChRs in Thy1-YFP mice, muscles were incubated for 2 h with 5 μg/mL Alexa 594 conjugated BTX (Molecular Probes). For immunostaining, entire muscles were dissected and either stained as whole-mounts or sectioned using a cryostat. Muscles were stained by first blocking overnight at 4°C (1% Triton X-100, 4% BSA in PBS) followed by incubation with Alexa 488- conjugated BTX and antibodies to neurofilaments (smi-312, Covance; 1∶500), SV2 (Developmental Studies Hybridoma Bank; 1∶10), or synaptotagmin-2 (znp-1; Zebrafish International Resource Center; 1∶250), or synaptophysin (Life Sciences; 1∶400) for at least 24 h in blocking solution. Muscles were washed for 3 h in PBS and incubated for 24 h with secondary antibody (Alexa-568 anti-mouse IgG1 or IgG2a and Alexa-647 anti-mouse IgG1; Molecular Probes). After washing for 3 h in PBS, muscles were whole-mounted on slides in Vectashield (Vector Labs).

### RNA extraction and RT-PCR

Total RNA was extracted using TRIzol reagent (Life Technologies). Small RNA was enriched and total RNA extracted using the *mir*Vana kit (Life Technologies). Following DNaseI digestion (Life Technologies), reverse transcription was performed using the AffinityScript kit (Stratagene) or High Capacity Reverse Transcription (Life Technologies). Semi-quantitative PCR and SYBR Green quantitative real-time PCR were performed using the following primers: pre-miR-133b-F, AGGCTTGGACAAGTGGTGCTCAA; pre-miR-133b-R, AAGGCTATGATGGCAAAACCAGC; pre-miR-206-F, GGAAGAAAGCAGCTTTTCCTTCTGC; pre-miR-206-R, GCCAAGGAACGAAGAAGTCAAC; pre-miR-133a-1-F (semi-quantitative), GGACATATGCCTAAACACGTGA; pre-miR-133a-1-F (real-time), GCTTTGCTAAAGCTGGTAAAATGG pre-miR-133a-1-R, GGTTGACAGTTGCTAGGTATTTGC; pre-miR-133a-2-F (semi-quantitative), GTCTGAATGTACATGTGACCCCTC; pre-miR-133a-2-F (real-time), GCTGAAGCTGGTAAAATGGAACC; pre-miR-133a-2-R, CACGTGACCTGGCTTTCTTG; pre-miR-1-1-F, GACTGAGACACAGGCGACAC; pre-miR-1-1-R, CATCGGTCCATTGCCTTTC; pre-miR-1-2-F, GCACTGGATCCATTACTCTTCC; pre-miR-1-2-R, GGAATGGGGCTGTTAGTATTACAG; AChRγ-F, CCAACCTCATCTCCCTGAATG; AChRγ-R, CAAGTTGATCTCACTGGTGCTG; Gapdh-F, CATGGCCTTCCGTGTTCCT; Gapdh-R, TGATGTCATCATACTTGGCAGGTT.

### Synapse enrichment

To obtain synaptic and extrasynaptic RNA fractions, P21 diaphragm muscle was dissected from thy1-GFP mice [30] and imaged under a fluorescence dissecting microscope (Leica). The synaptic region was clearly distinguished by the presence of fluorescent motor axons. Synaptic and extrasynaptic regions were dissected rapidly in ice-cold PBS, flash-frozen in liquid nitrogen and stored at −80°C until they were processed for RNA extraction and small RNA enrichment.

### LNA-DIG probes

miR-133b antisense and scrambled DNA oligonucleotides containing locked nucleic acid (LNA) modifications were ordered from Integrated DNA Technologies. LNA oligos were 3′-end labeled with digoxigenin (DIG) using the DIG oligonucleotide 3′-end labeling kit (Roche), according to the manufacturer's instructions. Unincorporated DIG was removed using sephadex Microspin G-25 columns (GE Healthcare), according to the manufacturer's instructions. Labeling efficiency was determined according to the 3′-end labeling kit instructions.

### miRNA Northern Blot

Northern blotting using LNA-DIG probes against mature miRNAs was carried out as previously described [50]. Briefly, 5–10 μg of total RNA or small RNA-enriched RNA was denatured in 2× loading buffer (80% formamide, 10 mM EDTA pH 8, plus bromophenol blue) at 70°C for 10 min and chilled on ice. 10 μl of a 10 bp DNA ladder (Life Technologies) was similarly denatured in 2× loading buffer. RNA samples and ladder were separated on a denaturing 15% polyacrylamide gel (50% urea, 0.5× TBE, 0.1% ammonium persulfate and 0.05% TEMED) in 0.5× TBE buffer at 200 V. The gel was stained with 4 μg/ml ethidium bromide in 0.5× TBE for 10 min. 78 nucleotide tRNA and 120 nucleotide 5S rRNA bands were imaged on an LAS3000 intelligent dark box (Fujifilm) and represent loading controls. RNAs were transferred in 0.5× TBE to a Nytran nylon membrane (Schleicher & Schuell) using a semi-dry transfer apparatus (Bio-Rad) at 15 V for 75 min. The membrane was air dried for 10 min and RNAs crosslinked to the membrane with 1200 mJ of UV light in a UV Stratalinker 2400 (Stratagene). Membranes were incubated with PerfectHyb Plus hybridization buffer (Sigma) for 30 min at 20–22°C below the melting temperature (Tm) of the LNA probe in a shaking water bath or a mini hybridization oven. Membranes were incubated with 0.1 nM of DIG-LNA probe in PerfectHyb Plus (1 pmol in 10 ml buffer) overnight at 20–22°C below the probe Tm. After probe hybridization, membranes were washed with 2×SSC+0.1% SDS for 30 min followed by 0.5×SSC+0.1% SDS at 30°C below probe Tm. In some cases, an additional, high stringency wash was performed using 0.1×SSC+0.1% SDS at 30°C below probe Tm. Membranes were washed in washing buffer (Tris-buffered saline +0.3% Tween) and blocked in 1× blocking solution (Roche, 10× stock diluted in washing buffer) for 30 min at room temperature. Membranes were incubated with an anti-DIG antibody conjugated to alkaline phosphatase (Roche) diluted 1∶10 000 in 1× blocking solution for 1 hour at room temperature. Membranes were washed three times with washing buffer, 15 min each, and finally washed twice with 0.1 M Tris-Cl pH 9.5+0.15 M NaCl, 15 min each. Membranes were placed on one sheet of a clear plastic sheet protector and covered with CSPD, ready-to-use (Roche) and a second clear plastic sheet. Luminescence at 477 nm was recorded by exposure of Hyblot CL autoradiography film (Denville Scientific) and subsequent development.

### Whole mount miRNA *in situ* hybridization

Whole mount miRNA *in situ* hybridization of P0 diaphragm muscle was performed as previously described for mouse embryos [31].

### SOD1(G93A) transgenic mice

miR-133b heterozygous mice were mated with transgenic mice overexpressing a mutant form of human Superoxide Dismutase (SOD1) under the control of the endogenous *SOD1* promoter [B6SJL-Tg(SOD1*G93A)1Gur/J; The Jackson Laboratory #002726] [48]. The resulting miR-133b het; SOD1(G93A) male offspring were mated to miR-133b heterozygous females. miR-133b WT, het, KO and miR-133b WT, het and KO; SOD1(G93A) mice were weighed each week from one month of age until they were sacrificed at a humane endpoint at which the animals could not right themselves within 15 sec of being placed on their side. Disease onset was retroactively determined as the age at which the mice reached their peak body weight. Disease progression was calculated as the duration between reaching peak body weight and when the animals were sacrificed. Disease progression was also followed by the hindlimb splay test and beam test. In the hindlimb splay test, each mouse was suspended by its tail for 5 seconds, at least 20 inches from any surface, and the resulting limb splay was scored arbitrarily based on the angle of the outstretched hindlimbs. A fully outstretched splay similar to that of healthy wildtype animals was given a score of 0; an acute splay angle where limbs were straight but angled towards the body was assigned a score of 1; animals whose hindlimbs were bent and tucked towards the body were given a score of 2; and animals whose hindlimbs were fully bent and touching the body were given a score of 3. In the beam-walking test, fine motor control was assessed by arbitrarily scoring the ability of the animals to navigate an elevated beam [51]. Each mouse was placed on the rim of an empty cage, with a box of food suspended on the opposite end of the cage to provide a goal. As the mouse moved across the rim of the cage, its foot placement was scored as follows: 0 =  normal (feet placed on rim of cage) 1 =  mildly impaired (uses sides of cage) 2 =  moderately impaired (barely able to walk) 3 =  severely impaired (is not able to walk and falls). Clinical onset was defined as the time at which the sum of the scores for splay and beam test reached a value of 3 without going below 3.

### Statistical analyses

All data were analyzed using Prism software (Graphpad Software, Inc.). Synaptic enrichment, synapse elimination, NMJ reinnervation, SOD1 data and morphological aging were analyzed by unpaired two-tailed t-test. Data are presented as means plus SEM.

## Supporting Information

Table S1 **Predicted targets of both miR-206 and miR-133b.** Target prediction algorithms were used to determine murine mRNA targets containing predicted binding sites in the 3'UTR for both miR-206 and miR-133b. The following websites were used: www.targetscan.org, www.microrna.org, www.mirdb.org, http://diana.cslab.ece.ntua.gr/microT/ (DIANA microT), and www.mirbase.org.
(XLSX)
